# Correcting motion-related B_0_ inhomogeneities in magnetic resonance imaging via combined spherical harmonics and AC/DC matrix coils using DL-based prediction–simulation study

**DOI:** 10.1038/s41598-026-56900-z

**Published:** 2026-06-16

**Authors:** Mohammad Khosravi, Wolfgang Bogner, Bernhard Strasser, Jason Stockmann, Christian Menard, Georg Langs, Günther Grabner, Beata Bachrata, Stanislav Motyka

**Affiliations:** 1 Department of Medical Engineering, Carinthia University of Applied Sciences, Klagenfurt, Austria; 2 Department of Biomedical Imaging and Image-Guided Therapy, High Field MR Center, Medical University of Vienna, Vienna, Austria; 3 Christian Doppler Laboratory for MR Imaging Biomarkers (BIOMAK), Department of Biomedical Imaging and Image-Guided Therapy, Medical University of Vienna, Vienna, Austria; 4 Harvard Medical School, Boston, MA USA; 5 Department of Radiology, A.A. Martinos Center for Biomedical Imaging, Massachusetts General Hospital, Charlestown, MA USA; 6 Comprehensive Center of Artificial Intelligence in Medicine (CAIM), Medical University of Vienna, Vienna, Austria; 7 Computational Imaging Research Lab, Department of Biomedical Imaging and Image-Guided Therapy, Medical University of Vienna, Lazarettgasse 14, Vienna, Austria

**Keywords:** Deep learning, B_0_ Inhomogeneities, Shimming, AC/DC matrix coils, Real-time shimming, Motion

## Abstract

MR data quality depends on static magnetic field (B_0_) homogeneity. At the beginning of each session, a brief field map quantifies subject-specific B_0_ variation, and shim coils are then set to counteract it. Conventional spherical-harmonic (SPH) shims have limited shimming power, motivating localized multi-coil (AC/DC) systems. However, subject motion can perturb the optimized field, necessitating real-time shim updates that require rapid tracking of B_0_ changes. We simulated real-time shimming under motion using jointly first-order SPH and a 31-channel AC/DC matrix coil. Measured B_0_ data initially shimmed with SPH were augmented with AC/DC terms in simulation, and real-time control was evaluated. Shimming with AC/DC coils added to the SPH coils improved field homogeneity, but motion eroded these gains. With simulated real-time updates informed by deep learning, B_0_ homogeneity was effectively maintained even during substantial motion. Performance matched simulated navigator-like real-time shimming with gradient-echo and echo-planar imaging, while adding no extra scan time in main imaging sequences. Multi-coil shimming offers clear benefits, but the gains may be reduced if shim terms are not updated in real time. Deep-learning-driven prediction of B_0_ changes provides a practical path to sequence-agnostic, motion-robust shimming across a broad range of MR protocols.

**Supplementary Information:**

The online version contains supplementary material available at https://doi.org/10.1038/s41598-026-56900-z.

## Introduction

Magnetic resonance imaging (MRI) provides noninvasive, high-resolution visualization of anatomical structures, especially soft tissues. A key requirement is high homogeneity of the static magnetic field (B_0_) within the imaging volume^1^. Differences in magnetic susceptibility between brain tissue, bone, and air lead to subject-specific spatial B_0_ inhomogeneities that cause image distortion, signal loss, and image blurring. In MR spectroscopy (MRS) and MR spectroscopy imaging (MRSI), B_0_ inhomogeneities lead to spectral line broadening, which decreases spectral resolution, as well as reduce the effectiveness of frequency-dependent pulses, such as those used for water suppression^2^.

The homogeneity of the B_0_ field can be adjusted using shim coils. B_0_ shimming methods can be categorized into two types: (i) static and (ii) dynamic. Static shimming involves fixing the shim coil currents throughout the entire MRI sequence^1^, whereas dynamic shimming involves updating the shims dynamically during different parts of the scan, e.g., dedicated shimming for each slice (or slab)^3–6^. In this study, static shimming is considered as whole-brain (WB) shimming, wherein the shim currents are applied uniformly for the entire brain. Conversely, dynamic shimming is implemented as slice-wise (SW) shimming, in which the shim adjustments are optimized independently for each slice.

To determine the shim coil currents that are required to homogenize the B_0_ field, it is necessary to know the subject-specific map of B_0_ inhomogeneity, or ‘field map’. Conventionally, field mapping is performed once before the scan despite the fact that the B_0_ field is usually not stable throughout the imaging process^7^. Long MR sequences are particularly sensitive to temporal B_0_ instability caused by subject motion^8,9^, but also by physiological processes such as respiration and cardiac pulsation^10–12^, and scanner instabilities. Real-time shimming approaches entail updating the shim coil currents continuously during the scan in order to adapt to temporal B_0_ changes^1^. However, the accuracy and swiftness of real-time B_0_ measurement remain a challenge.

The most common active shimming method is B_0_ shimming using spherical harmonic (SPH) coils, which are integrated into the MR scanner hardware^1^. Multi-coil shimming systems utilize numerous small, individually-controlled coils placed around the imaging area to provide a more localized response^7,13^. The added degree of freedom in the multi-coil corrective shim fields results in a superior correction of B_0_ inhomogeneities compared to lower-order SPH shim coils. This is particularly important in areas such as the orbitofrontal cortex, where strong local field distortions occur^14–17^ and at ultra-high fields. For instance, SW multi-coil shimming (with no real-time updating) was shown to reduce the EPI distortion compared to shimming with SPH coils of up to second order^18,19^. Higher-order SPH coils, including fourth-order implementations, can also reduce residual B_0_ inhomogeneities. However, multi-coil shim coils use low-inductance and low-cost local loops that require lower voltages, enabling faster switching and reducing eddy current effects compared to SPH shim coils^20^. Therefore, the advantages of multi-coil shimming cannot be overlooked. Recent advancements in multi-coil technology include AC/DC coils, which integrate two distinct functionalities within a single structure, enabling more compact and efficient coil design^19^.

Various approaches have been developed to obtain information on temporal B_0_ field changes during acquisition, including MR data-based techniques^21,22^ and external field monitoring techniques^23,24^. MR data-based methods, such as volumetric navigators (vNavs), track changes in the B_0_ field throughout the scan by alternating the primary sequence with a rapid EPI field mapping sequence^25–27^. However, the prolonged acquisition times and implementation difficulties with most imaging sequences are notable drawbacks of vNavs^21,28^. Other navigators, such as FID navigators, are easier to implement, but are limited to global frequency shifts or very low-resolution field maps, unsuitable for correcting spatially-localized B_0_ inhomogeneities^29^. Servo navigation enables prospective correction of motion and associated B_0_ variations, demonstrated for shimming with first-order SPH coils. While enabling efficient real-time updates, such approaches require sequence-specific implementation^30^. External monitoring techniques such as NMR probes^31^ function independently of the MR scanner, making them suitable for use with any imaging sequence. However, simulations of B_0_ fluctuations suggest that these probes are not sufficient for SPH coil configurations beyond the second order^32^. Furthermore, they are ineffective at detecting magnetic field variations originating from inside the imaging region, such as those caused by magnetic susceptibility effects^33^.

A newly emerging deep learning (DL)-based method for assessing real-time, motion-related variations in the B_0_ field^34^ requires only motion information (e.g., from an external tracking system or a navigator-based approach), eliminating the need for external field monitoring techniques or repeated measurements. This approach has been shown to provide B_0_ predictions that are comparable to those of EPI-based vNav-equivalent field maps, without extending sequence acquisition time. To improve prediction accuracy, the DL model is fine-tuned for each subject by using B_0_ field maps acquired at several motion positions at the beginning of the scanning session (as prescans). Although this slightly prolongs the total scan time, it does not prolong the primary acquisition sequence, nor require its adjustment.

This simulation study extends the DL-based B_0_ prediction approach to a combined SPH and AC/DC matrix coil and compares the performance of various shimming methods in addressing the motion-related B_0_ inhomogeneities. The residual B_0_ inhomogeneity and the spectral quality in MRSI are evaluated. Three shimming modes are compared: (i) WB shimming via SPH alone, (ii) WB shimming via a combination of SPH and AC/DC matrix coils, and (iii) SW shimming via a combination of SPH and AC/DC matrix coils. Additionally, real-time shimming using the SPH (zero and first order) and AC/DC coil configuration is simulated, and its performance when using the DL-based predicted field maps is compared with EPI-based vNav-like measurements and multi-echo GRE ground-truth scans.

## Material and methods

All procedures involving human participants were conducted in accordance with the Declaration of Helsinki and relevant local institutional policies. The study protocol was approved by the ethics committee of Medical University of Vienna. Written informed consent was obtained from all participants prior to their inclusion in the study.

### Acquisition of WB shimmed dataset using SPH coils

The B_0_ field maps and anatomical MP2RAGE images of 15 healthy volunteers (11 males and 4 females) were measured at a 7 T Magnetom + MR scanner (Siemens Healthineers, Erlangen, Germany)^34^. At the beginning of the acquisition session, WB shimming was performed using the scanner’s built-in (vendor-provided) algorithm, utilizing SPH shim coils up to second order (WB-SPH).

B_0_ field maps were acquired at 30 random positions, with the subjects performing head movements at their own discretion, to cover a possible, but still comfortable range within the head coil. Two B_0_ field mapping sequences were used:

1. 2D multi-echo GRE: nominal resolution = 1.9 × 1.9 mm, FOV = 240 × 240 mm, 80 slices with 2 mm thickness, and TA = 59 s. These acquisition parameters were chosen to match a standard GRE-based field mapping sequence.
2. 3D dual-echo EPI: nominal resolution = 8.0 × 8.0 mm, FOV = 256 × 256 mm, 32 slices with 8 mm thickness, and TA = 0.6 s. These acquisition parameters were chosen to resemble standard field mapping EPI-based vNavs.

For each head position, B_0_ maps were calculated from both the GRE sequence and the EPI sequence. The GRE-based B_0_ maps were calculated from the magnitude and phase images, coil combined by ASPIRE^35^, and phase unwrapped using ROMEO^36^. B_0_ maps from the dual-echo EPI sequence were calculated as Hermitian inner products^37^.

As an anatomical reference, MP2RAGE images were acquired for each subject at the initial position with the following parameters: nominal resolution = 1.1 × 1.1 mm, FOV = 220 × 220 mm, 80 slices with 1.1 mm thickness, and TA = 4:57 min. For the after-motion positions, the MP2RAGE scan at the initial position was transformed into the respective position by applying the transformation matrix from the co-registration of the 2D GRE magnitude images.

To ensure consistency in the inputs used for DL network training and evaluation, all images were downsampled in-plane to a matrix size of 128 × 128 × 80. The EPI-based B_0_ field maps were upsampled in the slice dimension to ensure consistency in evaluation.

### Preparation of semi-synthetic WB and SW shimming dataset including SPH + AC/DC coils

**Fig. 1:**
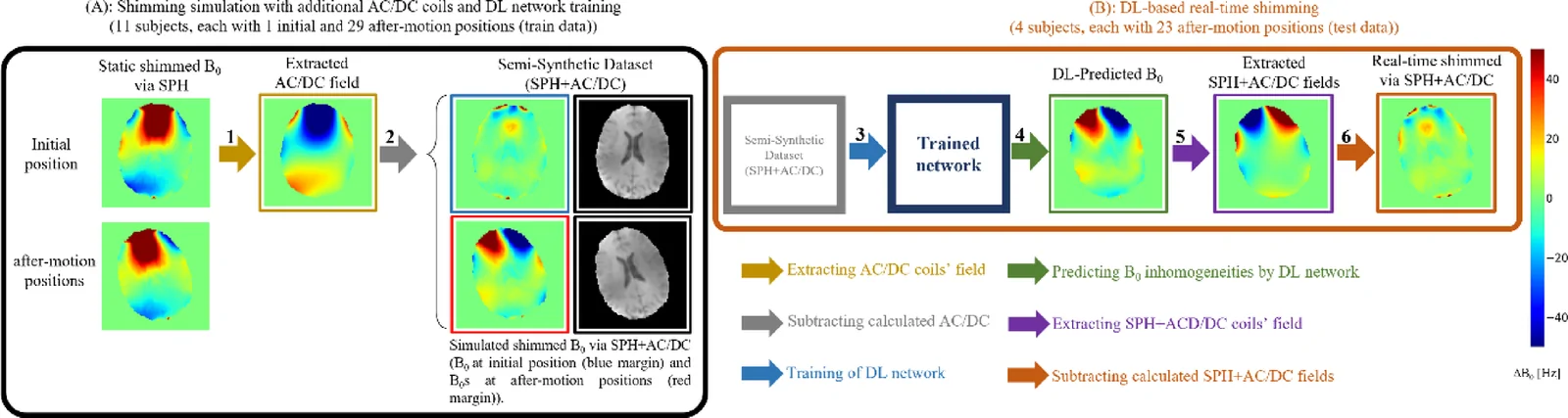
(**A**) B_0_ field map shimming simulation with additional AC/DC matrix coils: the AC/DC coil fields are extracted from the B_0_ field map at the initial position through shimming simulations (step 1, in the gold margin) and are then subtracted from all positions in order to create a new synthetic shimmed dataset combining SPH and AC/DC coils (step 2). The resulting B_0_ field map at the initial position (in the blue margin) along with anatomical MP2RAGE images at both the initial and after-motion positions (in the black margin) are utilized as the input for the DL network. The B_0_ field maps of the after-motion positions are considered as the ground truth for the DL network (in the red margin) (step 3). All steps are performed in two modes: whole-Brain (WB-SPH + AC/DC) and slice-wise (SW-SPH + AC/DC). (**B**) Simulated DL-based real-time shimming. The DL network is separately trained with WB-SPH + AC/DC and SW-SPH + AC/DC datasets and predicts B_0_ inhomogeneities at the after-motion positions (step 4, in the green margin). The predicted B_0_ maps are utilized to extract shim coils’ fields (step 5, in the purple margin) which are subtracted from B_0_ field maps at the after-motion positions. The result is the simulated DL-based real-time (WB and SW) shimmed dataset (step 6, in the orange rectangles).

In order to simulate DL-based real-time shimming via SPH + AC/DC matrix coils, the neural network needs to be trained with a dataset that consists of B_0_ field maps shimmed with both SPH and AC/DC coils. A semi-synthetic dataset, shimmed using both coil sets (SPH + AC/DC), was created from the acquired B_0_ fieldmaps by simulating additional shimming via 31-channel AC/DC matrix coils^19^ using MATLAB. The current limits for each individual coil (3.5 A) and for the total coil array (40 A) were adopted from previously reported values by Stockmann et al.^19^. As the AC/DC multi-coil configuration is not available at our research site, the AC/DC multi-coil fields utilized in this study were derived from measurements reported and publicly released by Stockmann et al.^19^. This simulation consisted of two steps: (1) calculating the AC/DC fields to achieve optimal B_0_ homogeneity based on the B_0_ field map at the initial position, (2) applying these estimated AC/DC fields to the initial and 29 other positions. This simulation was performed for both 2D multi-echo GRE and 3D dual-echo EPI B_0_ fieldmaps, under two regimes (see Fig. 1):

1. Whole-brain (WB): AC/DC fields were estimated for the whole brain at once and applied to whole brain B_0_ field maps in after-motion positions, resulting in a simulated WB shimmed dataset with SPH+AC/DC coil configuration (WB-SPH+AC/DC).
2. slice-wise (SW): AC/DC fields were estimated for each slice separately and applied to corresponding slices of B_0_ field maps in after-motion positions, resulting in a simulated SW shimmed dataset with SPH+AC/DC coil configuration (SW-SPH+AC/DC).

### Deep neural network

**Fig. 2:**
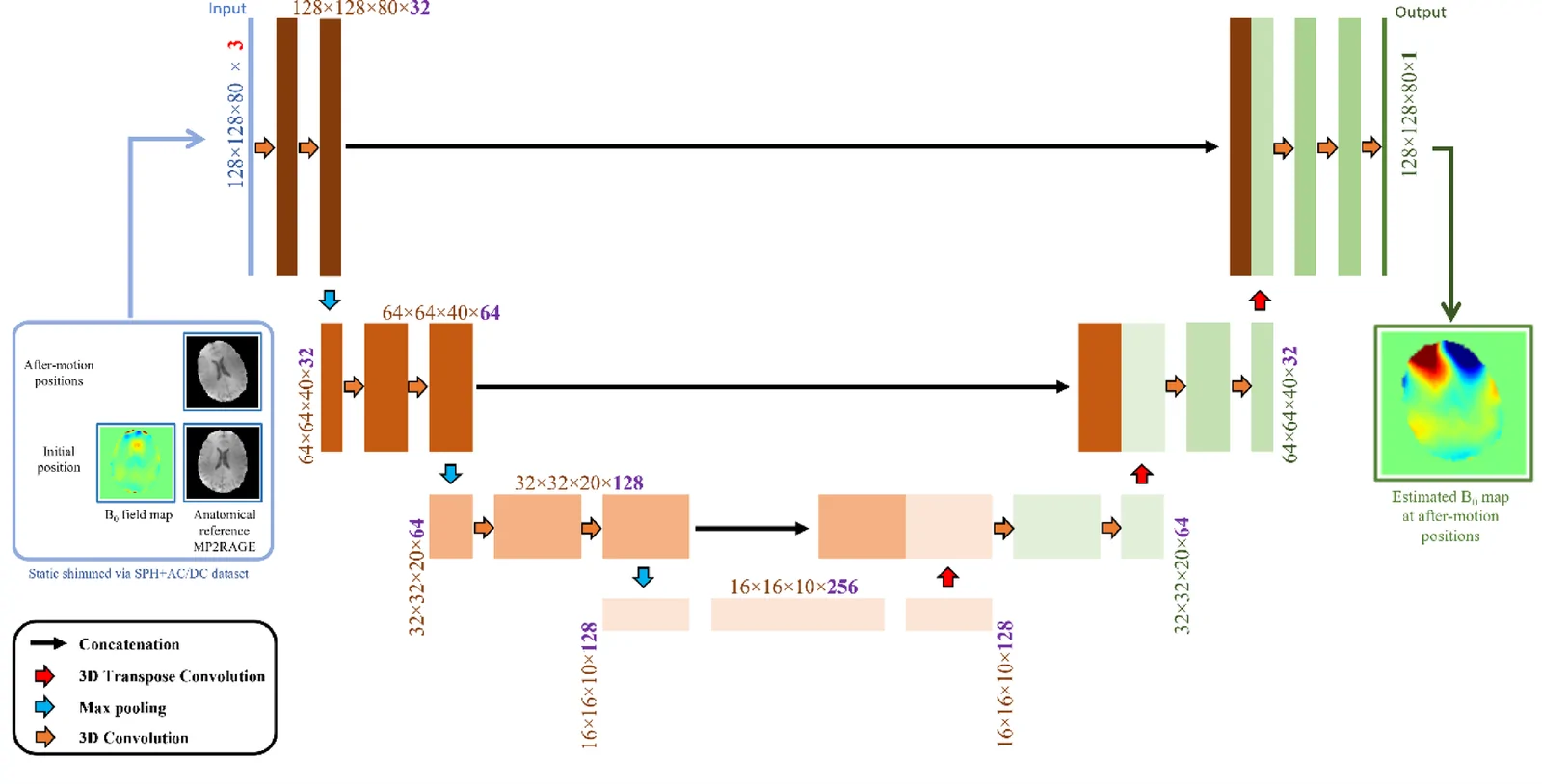
The proposed 3D U-net for real-time shimming simulation. The input (blue) for this network consists of 3 contrasts: B_0_ field map at initial position along with anatomical reference images at both initial and after-motion positions (in blue rectangle). All input images were resized to the resolution of 128 × 128 × 80 to ensure consistency across inputs. The output of the network is the B_0_ field map at the after-motion position, called predicted B_0_ field map.

The deep neural network architecture employed in this study was based on a U-Net architecture^38^. It consisted of three hierarchical levels in both the encoder and decoder paths, each level comprising two convolutional layers. All 3D convolutional layers employed a kernel size of 5 and a stride of 1, and were followed by a LeakyReLU activation function. The number of feature channels increased with network depth, specifically 32, 64, and 128 channels for the first, second, and third levels. The upsampling in the decoder path was performed using 3D transposed convolutional layers. Figure 2 shows the architecture of the U-Net proposed in this study.

The training process was conducted over 1000 epochs using a mini-batch size of 1. At each epoch, the training data were randomly permuted, followed by augmentation. Each training instance was augmented by randomly scaling up to second order SPH and AC/DC coils within physical limits. This network was optimized by an Adam (Adaptive Moment Estimation) optimizer^39^ with a learning rate of 1e−5 and weight decay of 1e−7, and with a loss function defined as the mean squared error between the ground truth B_0_ map and the predicted output.

Two separate instances of the neural network were trained on the newly generated synthetic datasets (WB-SPH + AC/DC and SW-SPH + AC/DC). Network training was performed in two stages: (i) pre-training and (ii) subject-specific fine-tuning. For pre-training, data from 11 subjects were selected for training and data from the other four subjects were reserved for testing. The training data included the GRE-based B_0_ field map at the initial positions along with anatomical MP2RAGE images at both initial and 29 after-motion positions. For the four test subjects, the pre-trained network was individually trained for 50 epochs per subject with the same DL model (Fig. 2). From the total of 29 after-motion positions, the first six were selected as input for the subject-specific fine-tuning, while the remaining positions were utilized for the assessment. Selecting the first six after-motion positions for fine-tuning was based on prior findings, where this approach yielded the best results in B_0_ prediction^34^. All simulated DL-based real-time shimming results are based on predicted B_0_ inhomogeneities obtained from subject-specific fine-tuned DL model.

### DL-based real-time shimming simulation using SPH + AC/DC coils

The trained models were utilized to estimate the B_0_ field maps for the after-motion positions. Based on these predicted B_0_ maps, the corresponding shim coil fields were calculated and subtracted from the after-motion B_0_ maps in order to simulate DL-based real-time shimming. In this work, shim updates were modeled using a combination of zero- and first-order SPH terms together with AC/DC coil fields. (Fig. 1B).

### Evaluation

The evaluation in this study consists of three parts:

#### Motion impact in WB and SW shimming using SPH and SPH + AC/DC coil setup

The impact of head motion on the B0 field without real-time correction (NC) was evaluated under three shim conditions: (i) WB-SPH, (ii) WB-SPH + AC/DC, and (iii) SW-SPH + AC/DC. The qualitative assessment was conducted by a visual comparison of the B_0_ field map at the initial position to the after-motion positions of the test dataset.

#### The evaluation of predicted B_0_ field maps via DL network

The evaluation of the DL-predicted B_0_ field maps (for both pre-trained and subject-specific fine-tuned DL networks) was compared to the ground truth GRE-based field maps. The evaluation was performed using a semi-synthetic dataset shimmed via SPH + ACDC in both WB and SW modes, as shown in the Supplementary Information.

#### Simulated real-time shimming performance using SPH + AC/DC

Evaluation of the simulated real-time shimming methods against no real-time correction, in both WB and SW modes. The following methods were compared: (i) No real-time Correction (NC), (ii) GRE-based simulated real-time shimming (RT-GRE), (iii) EPI-based simulated real-time shimming (RT-EPI), and (iv) subject-specific fine-tuned neural network-based simulated real-time shimming (RT-DL).

The qualitative assessment was performed on a representative B_0_ field map from the after-motion positions of the test dataset, and the quantitative evaluation included standard deviation and T_2_* effect analysis across four test subjects, each with 23 after-motion positions.

For all B_0_ field mapping methods, we calculated the standard deviation of the B_0_ field maps. Then, to evaluate the effects of motion-related inhomogeneities on T2* decay, the FWHM of synthetic MRSI data were investigated. Therefore, simple MRSI datasets were simulated by synthesizing a Lorentzian water signal in the FID domain (assuming T2 = 47 ms) for all voxels within brain masks created from the B_0_ maps. Then, the FID signals were frequency-shifted based on the intra-voxel B_0_ inhomogeneity. The synthetic MRSI datasets, with a voxel size of 1.9 × 1.9 × 2.0 mm^3^, were spatially downsampled in the k-space domain by a factor of four in each spatial dimension, resulting in MRSI data with a voxel size of 7.5 × 7.5 × 8.0 mm^3^. The FWHM maps of the downsampled MRSI data were calculated and data quality was assessed by computing the percentage of voxels exceeding the defined quality threshold (FWHM > 0.1 ppm)^40^, for each test subject individually. The impact of a B_0_ inhomogeneity of 0.1 ppm on MRS spectral quality is shown in Supplementary Fig. S1.

In addition, real-time shimming performance using SPH-only and AC/DC-only configurations was evaluated for the RT-DL approach to assess the individual contributions of each component (see Supplementary Information).

For all quantitative assessments, analysis of variance (ANOVA) was performed, followed by post hoc tests. Statistical significance is denoted as follows: *p* < 0.001 (***), *p* < 0.01 (**), and *p* < 0.05 (*).

## Results

### Motion impact in WB and SW shimming using SPH and SPH + AC/DC coil setup

**Fig. 3:**
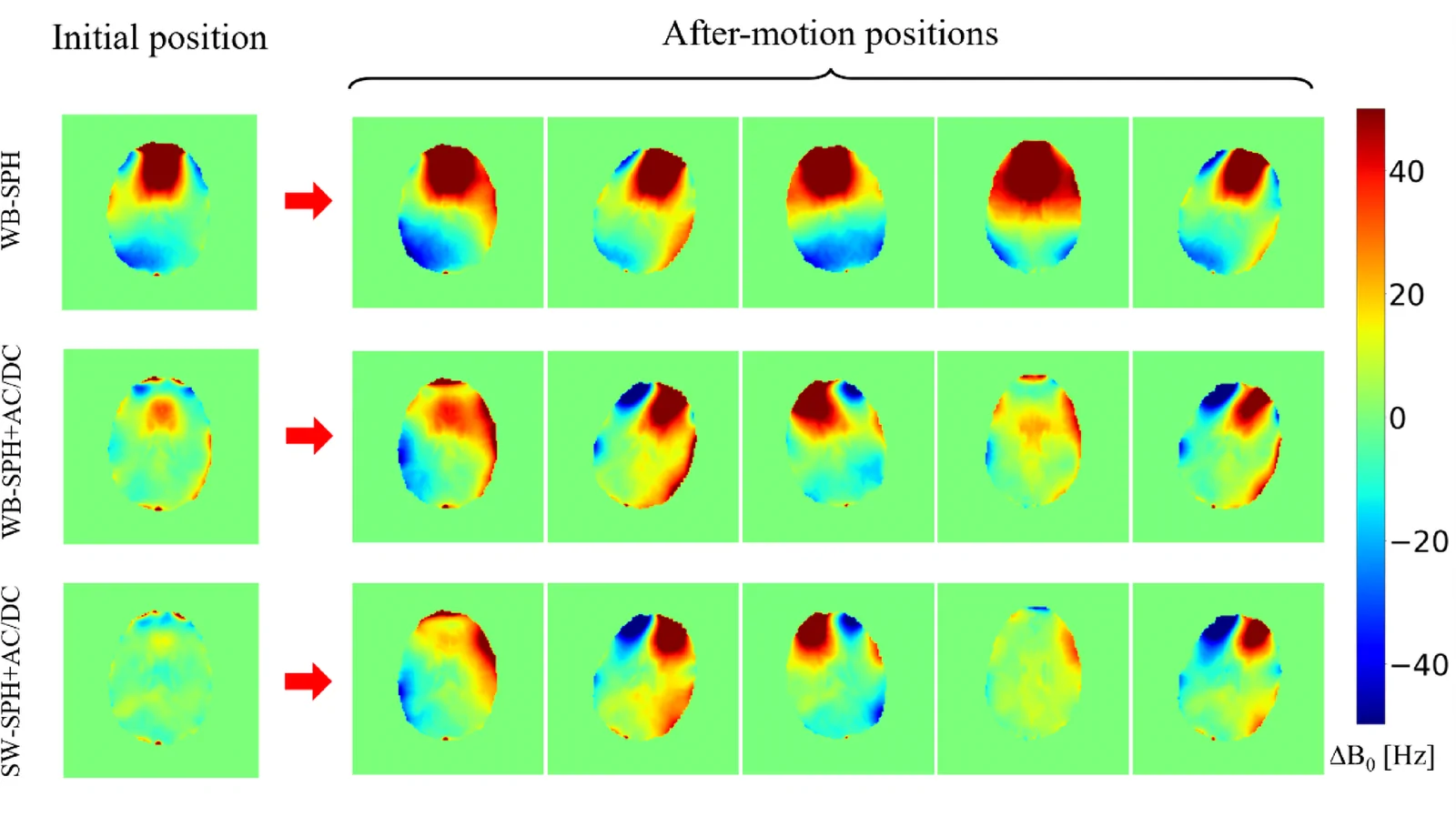
Shimming comparison under motion with no real-time correction (NC), depicted for exemplary after-motion test positions of a test subject. The B_0_ field map shimming using two different coil configurations: SPH (first row) and combination of SPH + AC/DC coils (second and third rows), in two regimes: whole-brain (WB) (second row) and slice-wise (SW) (third row). While shimming with SPH + AC/DC coils demonstrate better performance at the initial position (gray and white), it fails to address the effects of motion, disrupting B_0_ homogeneity in after-motion positions (dark blue and light blue).

A qualitative comparison was conducted to evaluate the performance of shimming with SPH coils in conjunction with AC/DC coils under motion (Fig. 3). Using additional AC/DC coils enhanced B_0_ field homogeneity at the initial position in both WB and SW shimming. However, since the shim currents were predetermined based on the initial position, B_0_ inhomogeneities increased following subject motion. Utilizing SPH coils alone resulted in strong remaining B_0_ inhomogeneities, even at the initial position. As shown in Fig. 3 (top row), these inhomogeneities were particularly pronounced in regions such as the frontal lobe, and were further exacerbated by subject motion. In contrast, shimming with the combined SPH + AC/DC configuration (Fig. 3, middle and bottom rows) reduced susceptibility-related inhomogeneities at the initial (pre-motion) position, with the most notable improvement observed in SW shimming mode. However, this improvement was compromised following subject motion, as susceptibility-induced inhomogeneities re-emerged in the after-motion positions.

### The evaluation of predicted B_0_ field maps via DL network

**Fig. 4:**
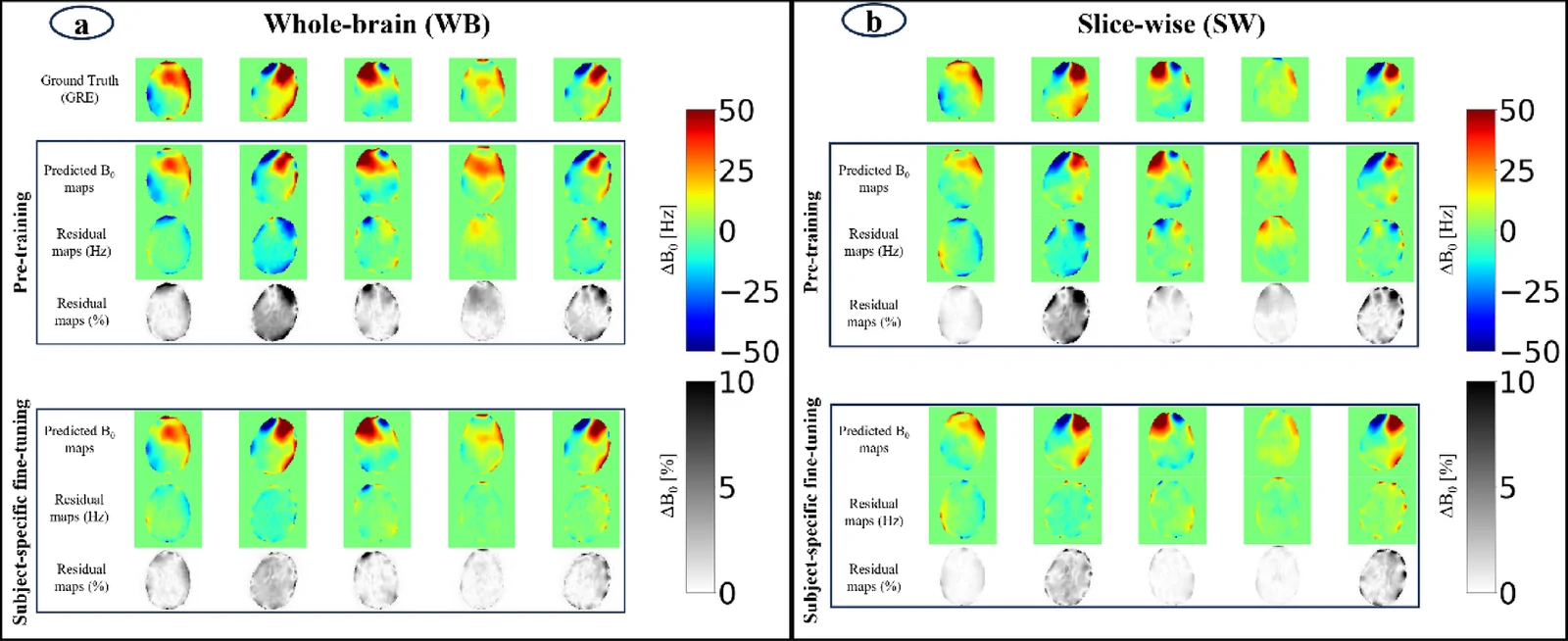
Comparison of predicted B_0_ field maps generated by the deep learning (DL) neural network in both pre-training and subject-specific fine-tuned stage with the ground truth GRE maps in after-motion positions for both (**a**): whole-brain (WB) and (**b**): slice-wise (SW) modes. At each stage, the first row shows the predicted B0 field map and second along with third rows display the corresponding error maps in hertz and percentage (relative to the ground truth), respectively. For the pre-training stage, the predicted B_0_ maps show low correspondence with the GRE maps, with considerable errors visible across multiple regions for both WB and SW modes. However, the predicted B_0_ maps in subject-specific fine-tune stage were closely match the GRE maps, although minor discrepancies remain.

For both WB and SW regimes, the predicted B_0_ field maps generated by the DL network in the pre-training step showed limited correspondence with the GRE-based field maps serving as the ground truth (Fig. 4). However, after subject-specific fine-tuning, these errors were significantly reduced (*p* value < 0.001, α = 0.05), as demonstrated visually in Fig. 4 and quantified (see Supplementary Fig. S2). Additional analysis comparing subject-specific fine-tuning using three versus six after-motion positions is provided (see Supplementary Fig. S2). The results indicate that increasing the number of fine-tuning positions improves prediction accuracy, with six positions yielding consistently lower residual errors.

### Simulated real-time shimming performance using SPH + AC/DC

**Fig. 5:**
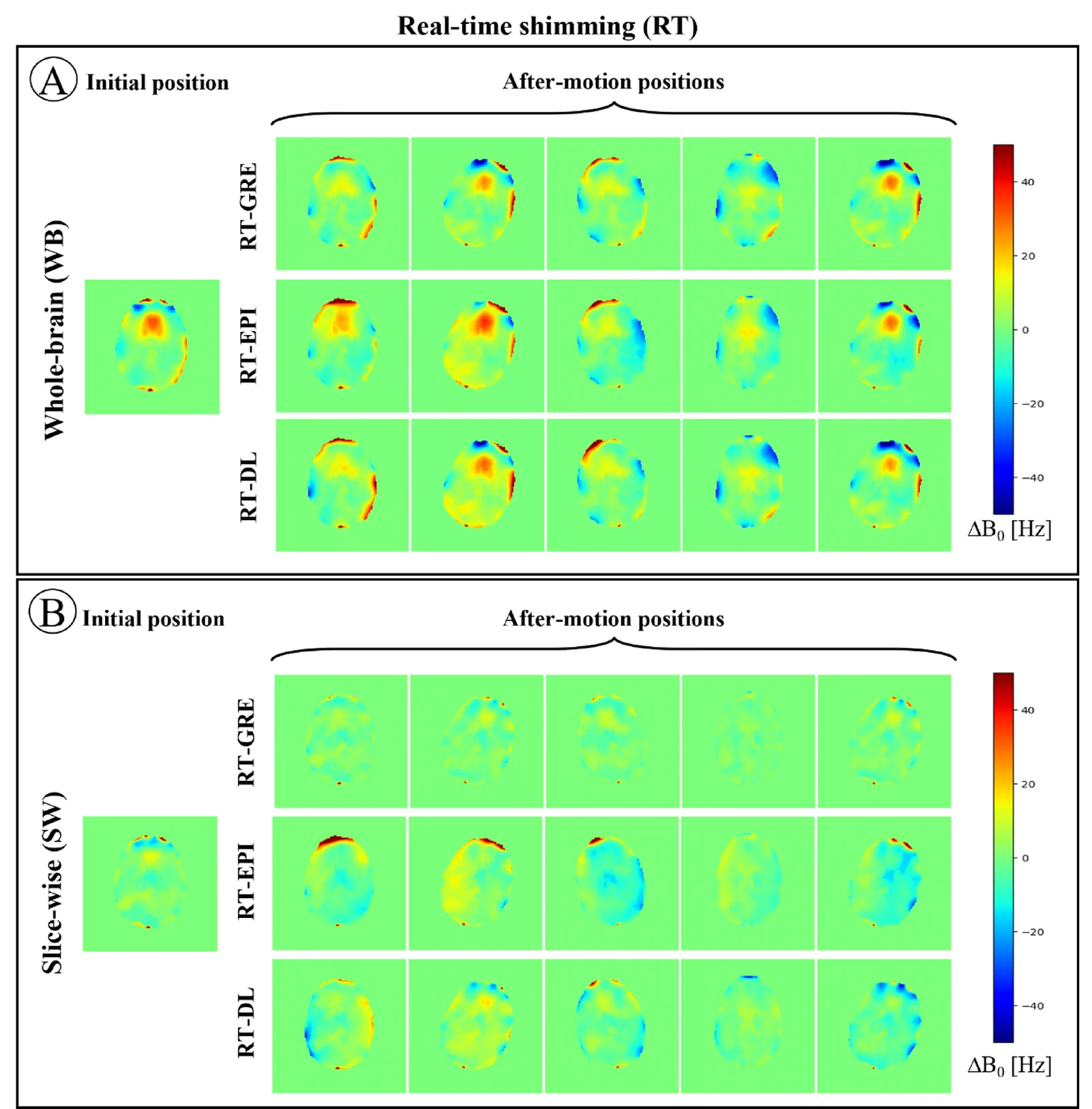
Shimming comparison under motion in simulated real-time shimming mode (RT) for an exemplary test subject using combined SPH + AC/DC coil configuration. Each panel shows ΔB_0_ maps for the different RT methods: RT-GRE, RT-EPI, and RT-DL (based on fine-tuned deep learning model), in two regimes: (**A**) whole-brain (WB) and (**B**) slice-wise (SW). For both WB and SW regimes, the simulated real-time shimming methods effectively address the effects of motion disrupting B_0_ homogeneity, maintaining the field homogeneity over various head positions, although minor residual inhomogeneities at the edges remained. Note that the same after-motion positions as in Fig. 3 are depicted.

**Fig. 6:**
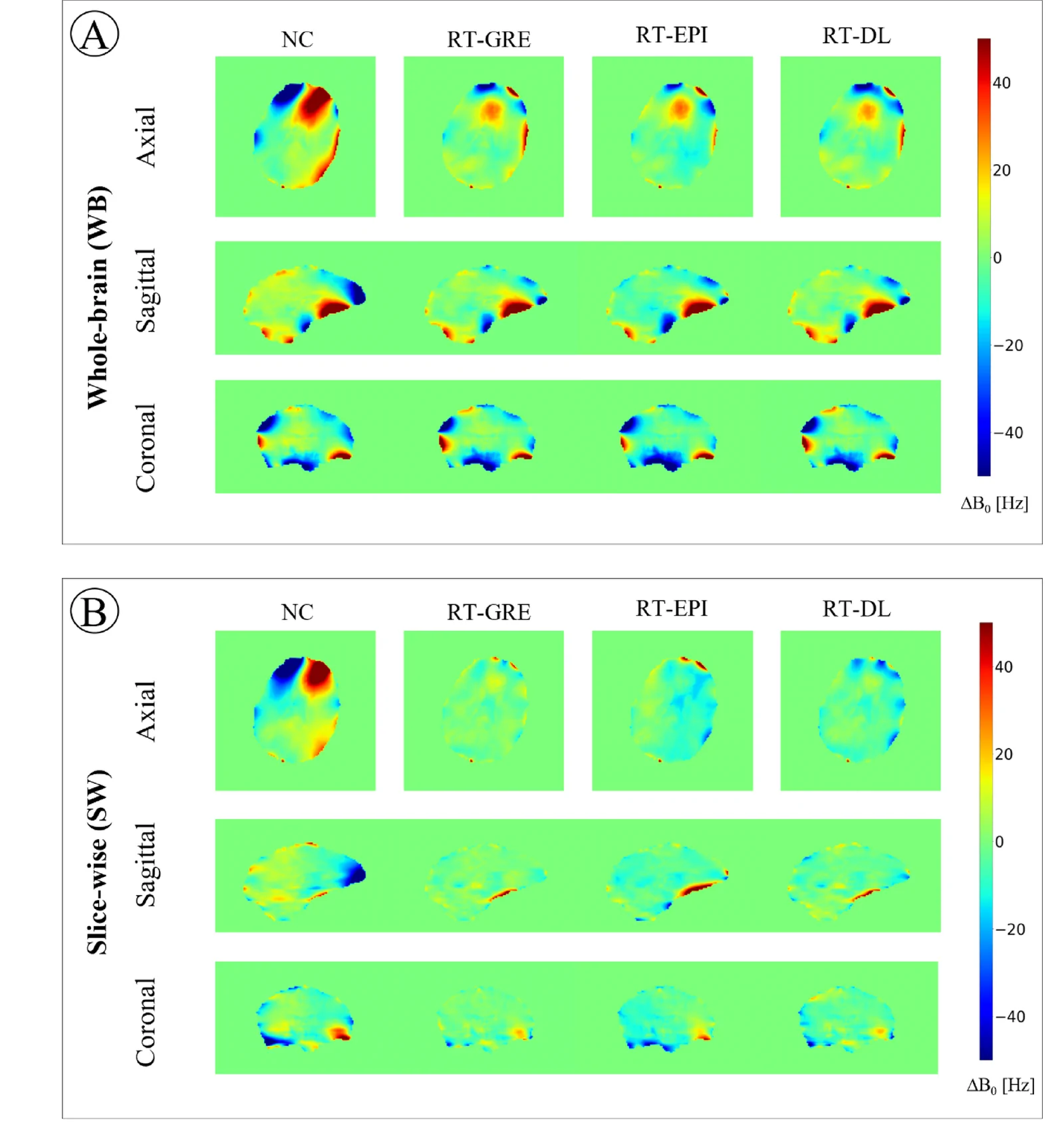
B_0_ field maps at an exemplary after-motion position shimmed using no simulated real-time correction (NC) and simulated real-time shimming with SPH-AC/DC coils. Simulated real-time shimming, whether whole-brain (WB) or slice-wise (SW), effectively maintains the B_0_ homogeneity despite the subject’s motion, in contrast to conventional shimming at both WB and SW modes, however, minor inhomogeneities at the edges remained. Shimming based on the subject-specific fine-tuned DL-predicted B_0_ field maps yield results comparable to the conventional GRE and EPI B_0_ measurement methods.

The qualitative comparison assessing the performance of simulated real-time shimming using the combined SPH + AC/DC configuration is presented in Figs. 5 and 6 for the test subjects, under two regimes: WB and SW. Figure 5 illustrates ΔB_0_ field maps of simulated real-time shimming methods in WB (A) and SW (B) regimes, shown for the same head positions as in Fig. 3. For both modes, simulated real-time shimming methods preserved B_0_ homogeneity across different head positions. Compared to the NC condition in Fig. 3, prominent inhomogeneities in the frontal lobe were reduced, with greater improvements observed in the SW mode. Figure 6 provides a detailed comparison of the RT methods versus NC across axial, sagittal, and coronal planes for a representative after-motion position. The simulation of RT shimming resulted in a marked reduction of motion-induced B_0_ inhomogeneities compared to NC for all shimming approaches, particularly in the occipital and parietal lobes. The reduction was more pronounced in the SW mode, highlighting the intrinsic advantages of slice-wise compared to whole-brain shimming. Nevertheless, both Figs. 5 and 6 demonstrate some residual inhomogeneities in WB and SW modes, particularly at the brain edges.

#### Standard deviation of B_0_ fieldmaps (NC vs. real-time shimming methods)

**Fig. 7:**
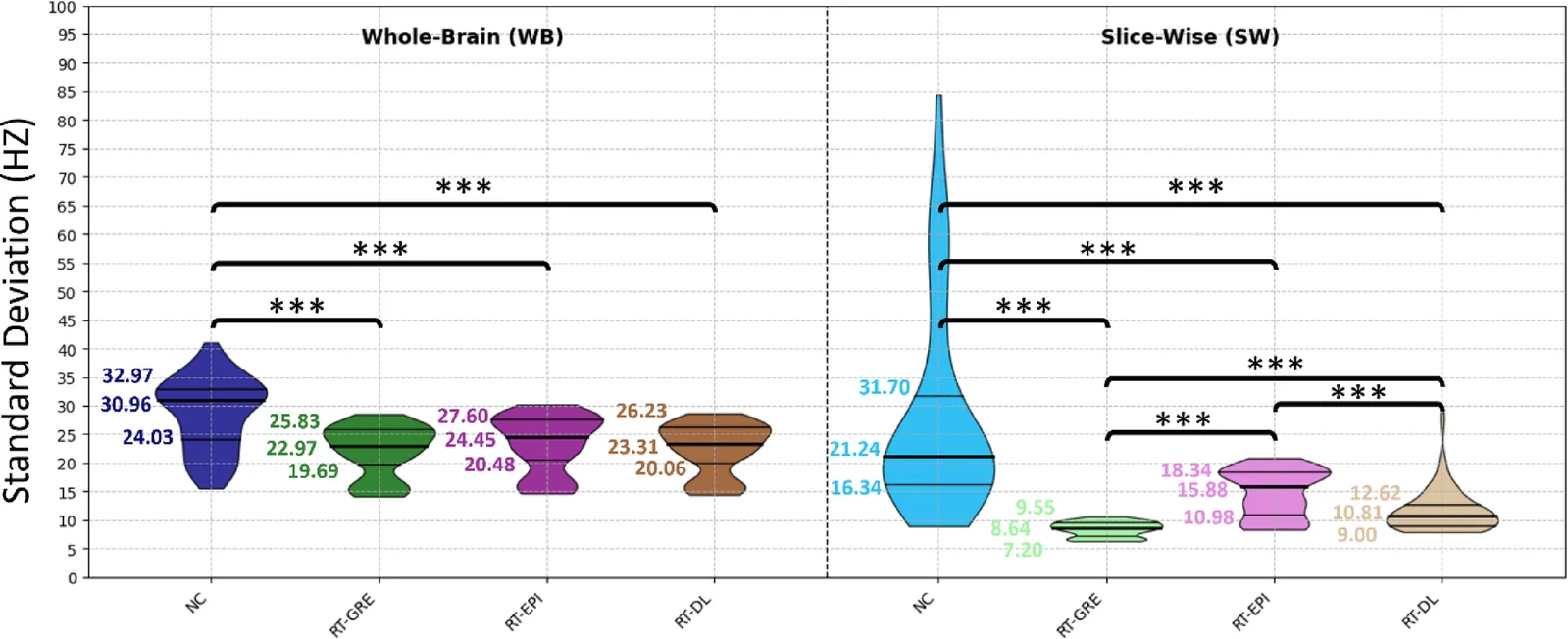
The standard deviation of B_0_ fieldmaps across four subjects and 23 motion positions per subject. Across both modes, all simulated real-time (RT) shimming methods (GRE-, EPI-, and subject-specific fine-tuned deep learning (DL-) based) significantly reduced B_0_ inhomogeneity compared to NC (*p value* < 0.001, α = 0.05). No significant differences were observed among the simulated real-time methods in the whole-brain (WB) mode. In the slice-wise (SW) mode, RT-GRE achieved significantly lower inhomogeneity than RT-EPI (*p value* < 0.01, α = 0.05). Notably, 75% of RT-DL values fell below 12.62 Hz, whereas only 50% of RT-EPI values were below 15.88 Hz, indicating a more favorable distribution for RT-DL.

Figure 7 shows the quantitative comparison of simulated real-time WB and SW shimming using the combined SPH + AC/DC configuration. It depicts the standard deviation of the B_0_ fieldmaps across 92 after-motion positions from the four test subjects (23 after-motion test positions for each subject). All simulated real-time shimming methods demonstrated a statistically significant improvement (*p* value < 0.001, α = 0.05) compared to the NC mode. In the WB mode (left), the median standard deviation of the B_0_ field maps for NC was 30.96 Hz, which decreased to 22.97, 24.45, and 23.31 Hz for RT-GRE, RT-EPI, and RT-DL, respectively. Among the simulated real-time methods, RT-DL exhibited a marginally lower median than RT-EPI, although these differences did not reach statistical significance. In the SW mode (right), the median standard deviation for NC was 21.24 Hz, which decreased to 8.64, 15.88, and 10.81 Hz for RT-GRE, RT-EPI, and RT-DL, respectively. Although RT-DL does not achieve the performance of RT-GRE, its overall distribution is substantially lower than that of RT-EPI, indicating a clear and statistically significant improvement (*p* value < 0.001, α = 0.05) over EPI-based shimming. Only in a limited number of positions does RT-EPI outperform RT-DL. Furthermore, 75% of RT-DL values fall below 12.62 Hz, whereas only 50% of RT-EPI values fall below 15.88 Hz.

Additional qualitative and quantitative results for SPH-only (zero and first order) and AC/DC-only real-time update configurations, evaluated within the RT-DL framework, are provided (see Supplementary Figs. S3 and S4). These results show that real-time updating using AC/DC coils yields substantially improved B_0_ homogeneity compared to SPH alone, while only minor differences are observed between AC/DC and combined SPH + AC/DC configurations.

#### Effects of motion-related inhomogeneities on MRSI spectral quality

**Fig. 8:**
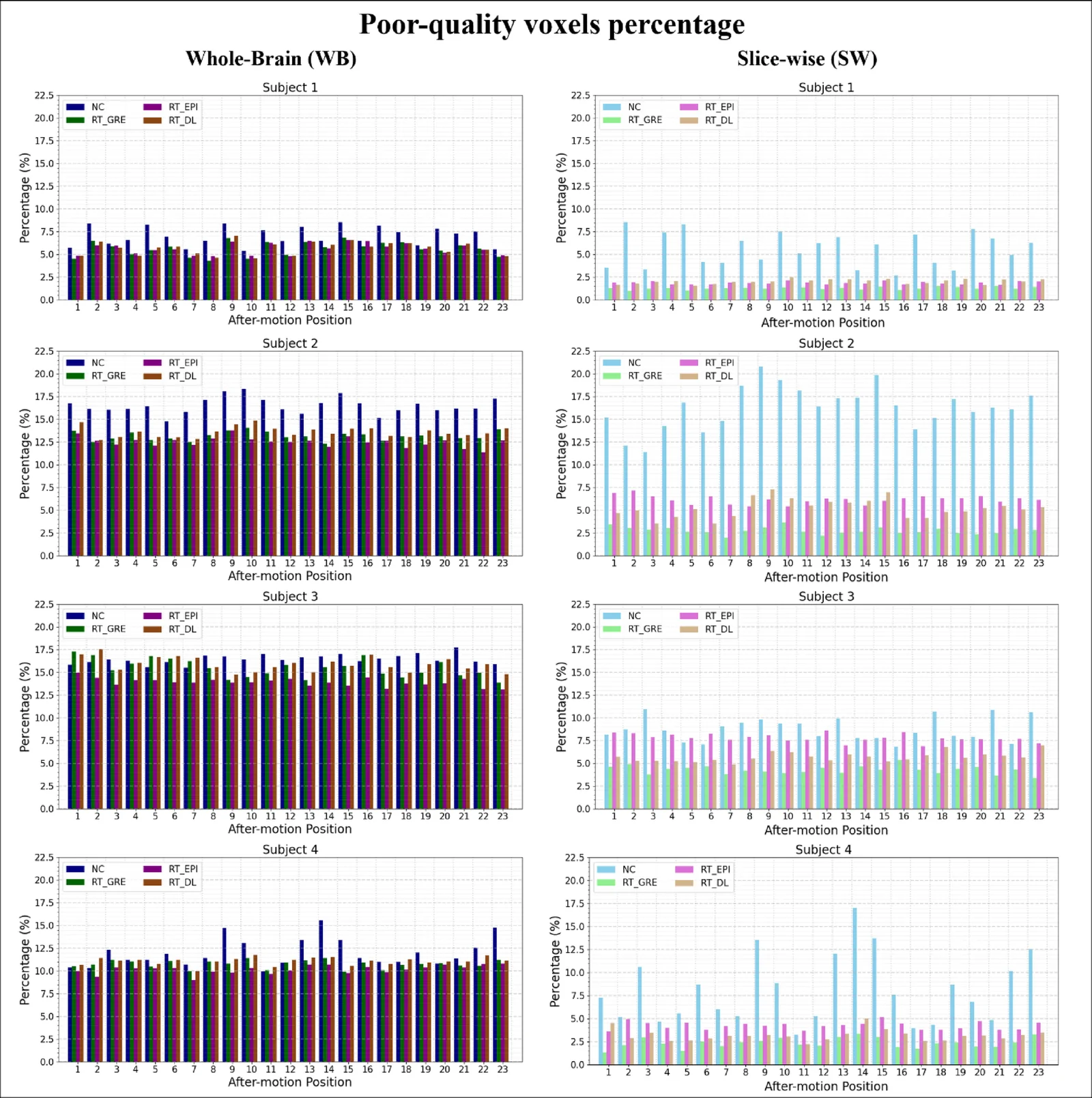
Comparison of poor-quality voxels percentage across four test subjects under different shimming conditions. Results are shown for whole-brain (WB) (left column) and slice-wise (SW) (right column) shimming modes, each evaluated across 23 motion positions. With no real-time correction (NC), motion consistently led to elevated rates of poor-quality voxels, whereas all simulated real-time methods (GRE-, EPI-, and DL-based) substantially mitigated these effects. The reductions were robust across subjects and motion amplitudes, with DL performing comparably to GRE- and EPI-based approaches.

Figure 8 shows the percentage of poor-quality voxels across the 23 after-motion positions for each of the four test subjects. For the NC condition, the percentage of poor-quality voxels for all subjects was higher compared to the simulated real-time methods, reflecting the impact of motion-induced B_0_ inhomogeneities. Simulated real-time shimming (GRE-, EPI-, and DL-based) not only substantially reduced poor-quality voxels but also maintained more stable levels across various motion positions. In SW mode (right), poor-quality voxel percentages over the 23 after-motion positions ranged between 3–8.5%, 11–21%, 7–11%, and 4–17% for NC, and decreased to 1.5–2.5%, 2.5–7.5%, 4–8%, and 2–5% with simulated real-time shimming using the combined SPH + AC/DC coil configuration for subjects 1–4, respectively. This indicates that the proportion of poor-quality voxels was both reduced and stabilized across positions. In WB mode (left), simulated real-time shimming likewise reduced the proportion of poor-quality voxels, though with slightly greater variability between positions. Performance differences among the three simulated real-time methods were minimal, with DL comparable to GRE- and EPI-based approaches.

## Discussion

This study evaluated the impact of motion on conventional and simulated real-time shimming using a combined SPH and AC/DC coil configuration. Incorporating multi-coil shimming alongside the conventional SPH shim coils was shown to improve B_0_ homogeneity particularly in and around the regions of high magnetic susceptibility, such as the frontal and temporal lobes^13,19,41–43^. However, because shim currents are set before scanning and held constant during acquisition, these improvements persist only in the absence of motion. As shown in the proposed simulation, real-time shimming, which involves adjusting the shim currents continuously during the acquisition, is essential for maintaining B_0_ field homogeneity in the presence of motion. This difference was shown to be even more prominent for real-time slice-wise shimming, in which the shimming is optimized for each slice separately, providing a highly localized response. As motion is unavoidable during real-world MR acquisitions, these findings indicate that the combination of multi-channel shim coils and real-time shimming is necessary to maintain the high B_0_ homogeneity critical for numerous applications.

Our results reinforce the importance of maintaining high B_0_ homogeneity for MRSI quality, as previously emphasized by Wilson et al., who suggested a FWHM > 0.1 ppm threshold to exclude poor-quality spectra^40^. The impact of B_0_ inhomogeneity on MRSI is consistent with earlier reports demonstrating reduced performance of water and lipid suppression methods which happens with short-TE acquisitions^40,42^. Increasing TE can partially compensate for poor water and lipid suppression but it inevitably leads to reduced signal, especially for metabolites with short T_2_ relaxation times and/or J-modulation^40^. Susceptibility of MRSI acquisitions to B_0_ inhomogeneities is particularly pronounced in anatomically challenging regions such as the anterior cingulate cortex, where 2nd-order SPH coils lack sufficient homogenization capability^40^. Kumaragamage et al.^42^ have demonstrated the benefit of multi-coil arrays over SPH coils for improving B_0_ uniformity in 3D MRSI acquisitions, but did not consider the impact of motion. In this simulation study, we analyzed the impact of motion in the absence of real-time shimming using combined coils (SPH + AC/DC). As depicted in Fig. 8, our findings showed that motion-related B_0_ inhomogeneities had a strong impact on the proportion of poor-quality voxels when using SPH + AC/DC coils, underscoring the necessity for real-time correction. In MRS, a substantial line broadening of spectra leads to loss of spectral resolution and potentially diagnostic errors. For instance, metabolites such as N-acetylaspartylglutamate (NAAG) and glutamate–glutamine (Glx) could become undetectable, while peaks such as myo-inositol (mI) and glutathione (GSH) could overlap to the extent that they could not be reliably distinguished. The simulation showed that a real-time shimming with the combined SPH and AC/DC coil configuration can markedly reduce the proportion of poor-quality voxels of MRSI data in the presence of motion. Overall, our findings indicate that real-time shimming via multi-coil setup could extend the feasibility of high-resolution whole-brain MRSI, including in regions previously considered unsuitable. Motion-related B_0_ inhomogeneities also affect the localization of voxels in single-voxel spectroscopy which have been analyzed by^26^ and not simulated in this study.

This simulation study also compared the theoretical real-time shimming performance of different B_0_ field mapping methods (i.e., DL-based prediction vs. actual measurements). The deep-learning approach to predicting motion-related changes in the B_0_ field^34^ was extended to incorporate shimming with an additional AC/DC matrix. This dataset was then used to train a 3D U-Net-based neural network, predicting motion-related B_0_ field changes in the combined SPH and AC/DC shimming mode. This enabled the simulation of real-time shimming. In WB mode, no significant difference in the standard deviation of the B_0_ fieldmaps was observed among the simulated real-time shimming approaches. Regarding the SW mode, the performance of RT-EPI was inferior to that of RT-GRE, likely due to its greater slice thickness, distortion, and increased noise levels. The RT-DL approach, while significantly improving over RT-EPI, did not reach the performance of RT-GRE and remained significantly different from the ground truth. A small number of RT-DL positions exhibited elevated standard deviation values, which may be attributed to the limited size and variability in the training dataset. The superior performance of the deep learning network compared to EPI may be attributed to the DL network being trained using the GRE-based ground truth data with a higher spatial resolution and reduced susceptibility-induced distortions. These results strengthen the proposed DL-based real-time shimming framework and expand the proof-of-principle implementation to the combined SPH and AC/DC coil configuration. The prediction step after fine-tuning requires less than 5 ms per position. Our approach performs similarly to having ground truth GRE-based B_0_ field maps (in WB mode) without requiring additional acquisition time in the main imaging sequence, if an external method for estimating subject movement, such as optical tracking is available. Therefore, it provides greater flexibility in applications across different MR sequences than navigator-based techniques, since even the EPI vNav requires 378 ms^44^, which substantially increases the TR of most parent sequences.

The simulated real-time shimming incorporating AC/DC shim coils effectively reduced motion-related B_0_ inhomogeneities. However, some residual inhomogeneities remained near the brain edges, as observed in Figs. 5 and 6. These are likely attributable to the strong magnetic susceptibility effects at tissue–air interfaces. According to previous findings on multi-coil shimming (Stockmann and Wald 2018), increasing the number of shim channels can further mitigate such edge-related inhomogeneities^20^. In our study, simulations were conducted using 31 coils; thus, it is expected that employing a higher number of channels would further diminish the remaining inhomogeneities.

The pre-trained network showed deficiencies in capturing individual anatomical variability and motion-specific inhomogeneities for unseen data, resulting from the limited training data size. By fine-tuning the network for each subject, it was able to capture unique anatomical features, resulting in more accurate B_0_ predictions and improved performance in simulated real-time shimming. This is consistent with a previous study^34^ which similarly demonstrated that subject-specific fine-tuning enhances the accuracy of DL-based B_0_ prediction. Currently, subject-specific fine-tuning requires six additional multi-echo GRE-based B_0_ map acquisitions (59 s each), followed by approximately 1.5–2 min of post-acquisition network fine-tuning. To avoid the need for fine-tuning, the dataset could be further enhanced by acquiring data from a larger number of subjects and motion positions. Alternatively, novel network architectures with better generalizability could be explored in the future.

Augmented coil maps were generated by assigning randomly scaled measured basis field values constrained within the range of the experimentally measured shim coil maps, ensuring that the simulated fields remained physically realistic. However, other augmentation strategies were not considered. It should also be noted that the present augmentation was applied only within the defined ROI. Another potential limitation that may have decreased the model generalizability or its overall performance is the neglect of physiological fluctuations and scanner instabilities. While the physiological fluctuations, such as changes in the blood oxygenation, blood flow, and respiration-induced chest volume changes, are known to influence the B_0_ field in the brain^10–12,45^, they are usually neglected in shimming due to their rapid changes and typical field variations of only a few Hz. The scanner instabilities lead to a global frequency drift of typically less than one Hz per 5 min acquisition time^46^, thus also having only a minor influence on shimming.

This study was conducted using simulations, which allowed systematic comparison of the different shim modes and assessment of the method’s theoretical performance on the same data. The simulation of AC/DC matrix coil shimming was performed using realistic basis fields of a real 31-channel coil^19^ to approximate experimental conditions. While the data used in this study were acquired *in vivo*, the proposed real-time shimming approach was evaluated in simulation due to the unavailability of an AC/DC matrix coil at our research site. Consequently, the proposed method was evaluated under simulation conditions using semi-synthetic data generated from previously acquired *in vivo* measurements^34^. Evaluation using actual *in vivo* data remains an essential direction for future work. While the exact performance of the different shim modes may differ if a different multi-channel shim coil configuration was used, the general trends are expected to remain the same.

## Conclusion

This study focused on comparing the performance of various shimming methods in addressing motion-related B_0_ inhomogeneities, using a comprehensive simulation approach. The inclusion of AC/DC matrix shim coils was shown to improve B_0_ field homogeneity but may be influenced by subject motion, underscoring the need for real-time shimming. The DL-based approach for predicting motion-related B_0_ field changes^34^ was extended by incorporating AC/DC matrix shim coils to enable simulations of real-time shimming in a combined SPH and AC/DC coil configuration. The DL-based real-time shimming demonstrated comparable performance to shimming based on GRE ground truth and EPI navigator-like field maps, particularly in WB mode, offering a viable approach to real-time shimming. Successful implementation is expected to substantially preserve B_0_ homogeneity, mitigate motion-related inhomogeneities—especially in regions with high magnetic susceptibility effects—and ultimately improve the accuracy and reliability of clinical imaging.

## Supplementary Information

Below is the link to the electronic supplementary material.


Supplementary Material 1


## Data Availability

The datasets generated during and/or analysed during the current study are available from the corresponding author upon reasonable request.
