# Supplementary material for: Correcting motion-related B0 inhomogeneities in magnetic resonance imaging via combined spherical harmonics and AC/DC matrix coils using DL-based prediction–simulation study
Source: Sci Rep. 2026 Jun 16;16:27487. doi: 10.1038/s41598-026-56900-z (PMC13534436; doi:10.1038/s41598-026-56900-z)
Supplement: Supplementary file 1 [file 41598_2026_56900_MOESM1_ESM.pdf]

Supplementary Information

The impact of B_0 inhomogeneity on MRS spectral quality

Magnetic field imperfections can significantly affect the quality of magnetic resonance spectroscopy (MRS) signals by altering the shape and clarity of spectral peaks. This effect is illustrated in the Figure S1, where the spectrum without inhomogeneity (blue) shows sharp, well-defined peaks for metabolites such as NAA, creatine, and choline, while the spectrum with a 0.1 ppm inhomogeneity (red) displays broader and less intense peaks. As a result, neighboring signals begin to overlap, making it more challenging to clearly resolve and accurately quantify individual metabolites.

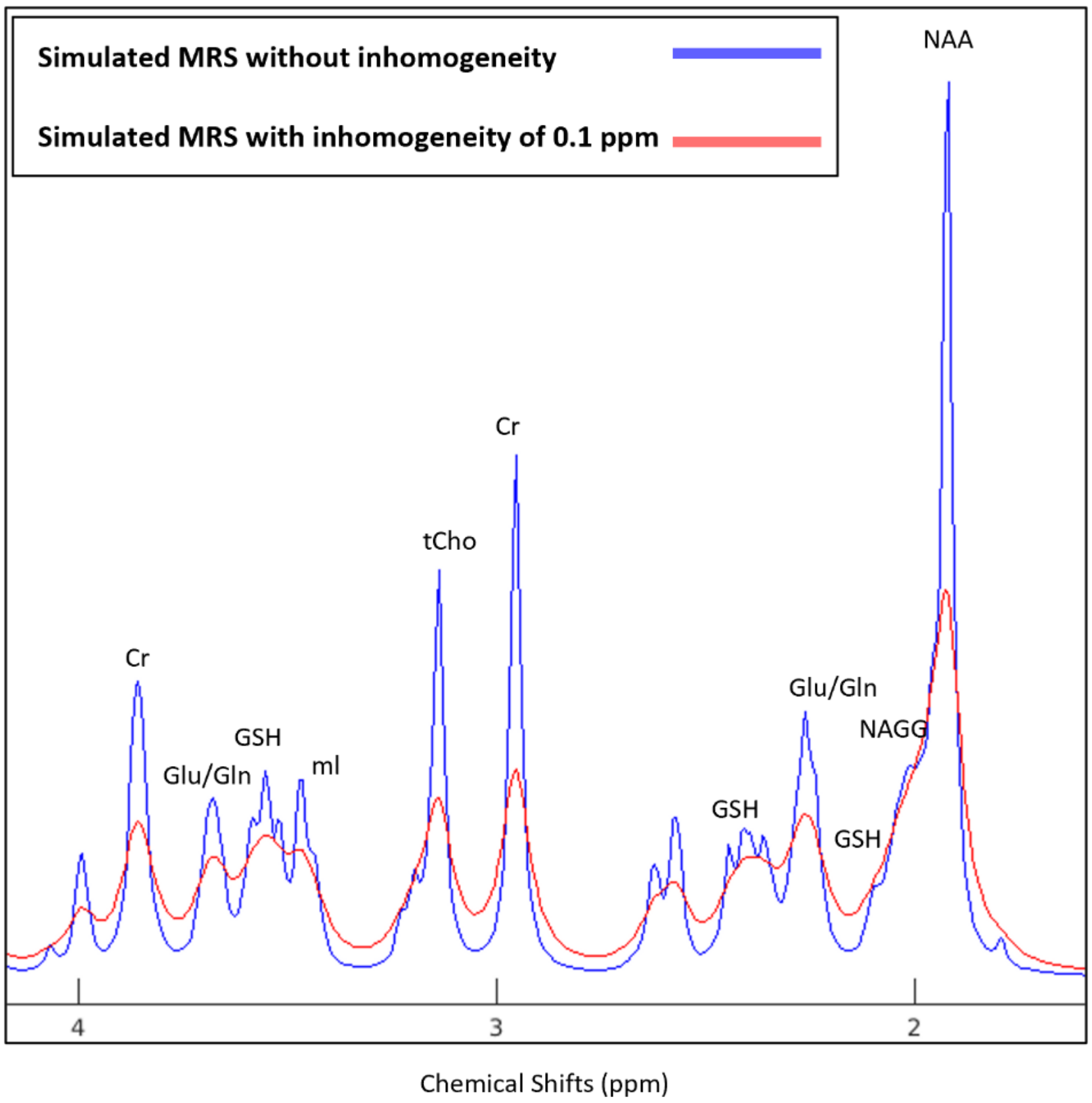

Figure S1. The impact of B_0 inhomogeneities (0.1 ppm or 30 Hz at 7 Tesla) on simulated MRS data.

Quantitative Evaluation of Predicted B_0 Field Maps (SPH+AC/DC)

The quantitative evaluation of differences between the predicted B_0 field maps and ground truth (GRE) B_0 field maps is presented in Figure S2. In addition to the primary approach based on fine-tuning with six after-motion poses (6p), we evaluate the performance of fine-tuning using only the first three after-motion poses (3p) to assess the effect of reduced subject-specific data. The results show that both 3p and 6p fine-tuning substantially reduce residual variability compared to pre-training. However, 6p consistently achieves lower SD values than 3p across both whole-brain and slice-wise modes, indicating improved accuracy with additional after-motion poses. This trend is particularly pronounced in the slice-wise mode, where higher variability is observed in the pre-trained model. Statistical analysis confirms significant differences between the different fine-tuning modes.

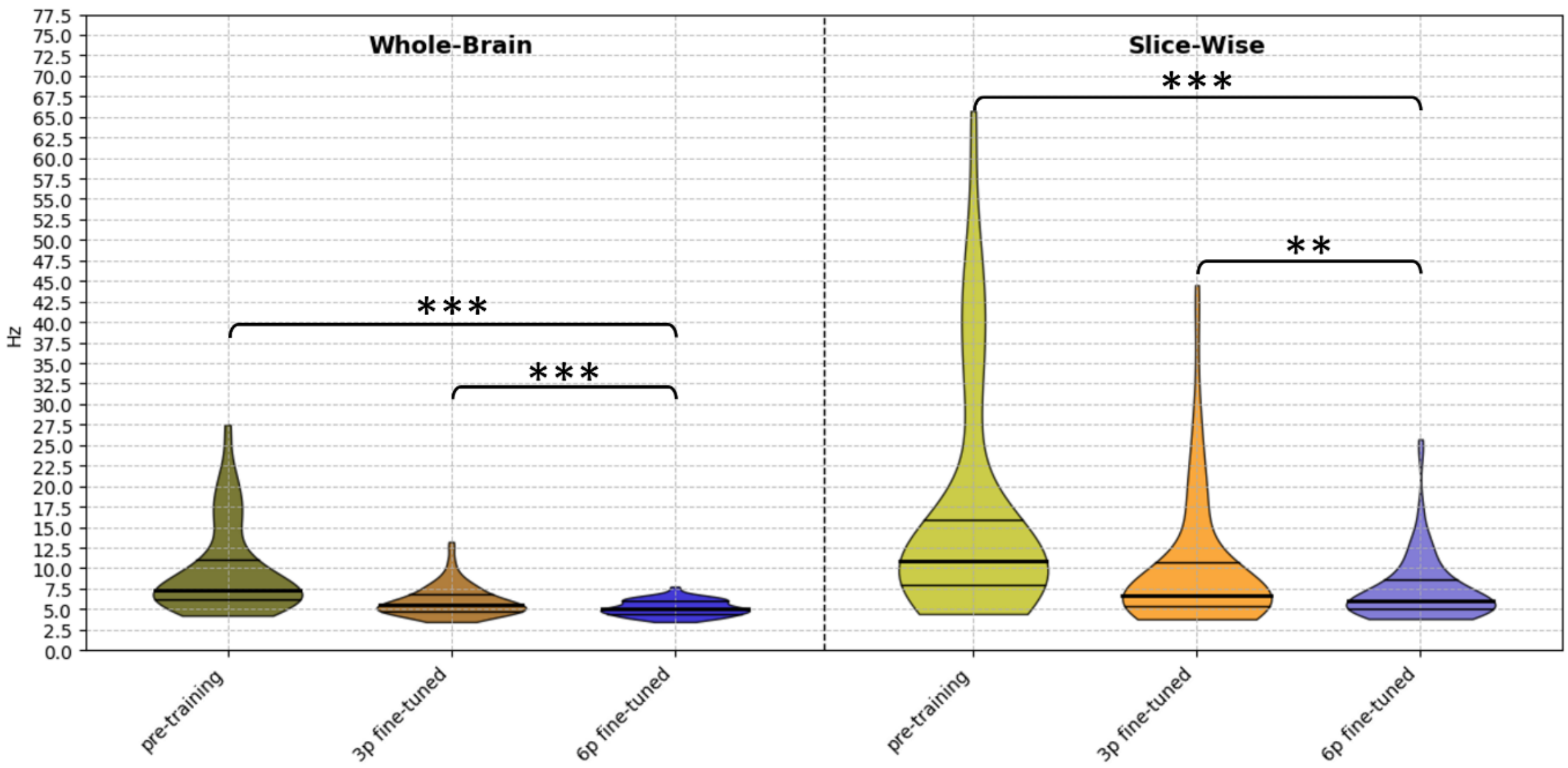

Figure S2. Standard deviations (SD) of the residual maps between the predicted B_0 field maps and the ground truth GRE maps for both whole-brain (WB) and slice-wise (SW) regimes across pre-training, 3-position (3p) fine-tuning, and 6-position (6p) fine-tuning. In both WB and SW regimes, subject-specific fine-tuning significantly reduced the SD of the residual maps (p-value < 0.001), with 6p fine-tuning achieving lower SD values than 3p (p-value < 0.01 in SW and p-value < 0.001 in WB).

Motion Analysis

Subject motion was quantified using rigid-body transformations relative to the initial pose and summarized in terms of translational displacement (mm) and rotational deviation (degrees), along with the standard deviation (std) and the coefficient of variation (CV) to assess relative variability. In this study, data from 15 subjects were analysed, comprising 319 after-motion samples for training and 92 after-motion samples for testing. As shown in Table S1, the overall mean translational displacement was 6.84 mm for the training data and 7.31 mm for the test data, with values ranging from 0.69 mm to 21.24 mm and from 1.97 mm to 10.36 mm, for the training and test data respectively. The mean rotational motion was 11.18° for the training dataset and 9.25° for the test dataset, with corresponding ranges of 0.34° to 41.53° and 2.02° to 21.43°. The CV values indicate moderate variability in rotational motion, while translational motion showed lower variability, particularly in the test dataset.

		Translation (mm)					Rotation (degree)				
	Number of samples	mean	std	min	max	CV	mean	std	min	max	CV
Train	319	6.84	3.88	0.69	21.24	0.57	11.18	7.99	0.34	41.53	0.71
Test	92	7.31	1.85	1.97	10.36	0.25	9.25	4.28	2.02	21.43	0.46

Table S1. Summary of motion statistics across the training and test datasets

Evaluation of deep-learning-based real-time shimming with different coil configurations

To further evaluate the contribution of different shim systems in real-time correction, additional shimming configurations were investigated. While the main analysis considers combined SPH (zero and first order) and AC/DC coil updates, additional simulations were performed to extract their respective roles. Specifically, real-time shimming based on DL-predicted B_0 field maps (RT-DL) was applied under three configurations: (i) AC/DC coils combined with zero and first-order SPH adjustments, (ii) AC/DC coils only, and (iii) zero and first-order SPH adjustments only in both whole-brain (WB) and slice-wise (SW) modes (Figure S3 and S4).

A qualitative comparison of the B_0 field maps after real-time shimming is shown in Figure S3. In the WB mode, the SPH-based shimming exhibits pronounced residual field inhomogeneities, particularly in inferior and peripheral regions, with large positive and negative deviations. In contrast, both AC/DC and combined SPH+AC/DC configurations substantially reduce these spatial variations, resulting in a more homogeneous field distribution across the brain.

In the SW setting, this improvement is even more evident. The AC/DC-based approaches effectively suppress localized field distortions and reduce the magnitude of extreme deviations, leading to a more uniform B_0 distribution compared to SPH alone. The visual difference between AC/DC and combined SPH+AC/DC is minimal.

Overall, the qualitative results confirm that incorporating AC/DC coils enables more effective correction of spatially complex B_0 inhomogeneities, particularly in regions where conventional SPH-based shimming remains insufficient.

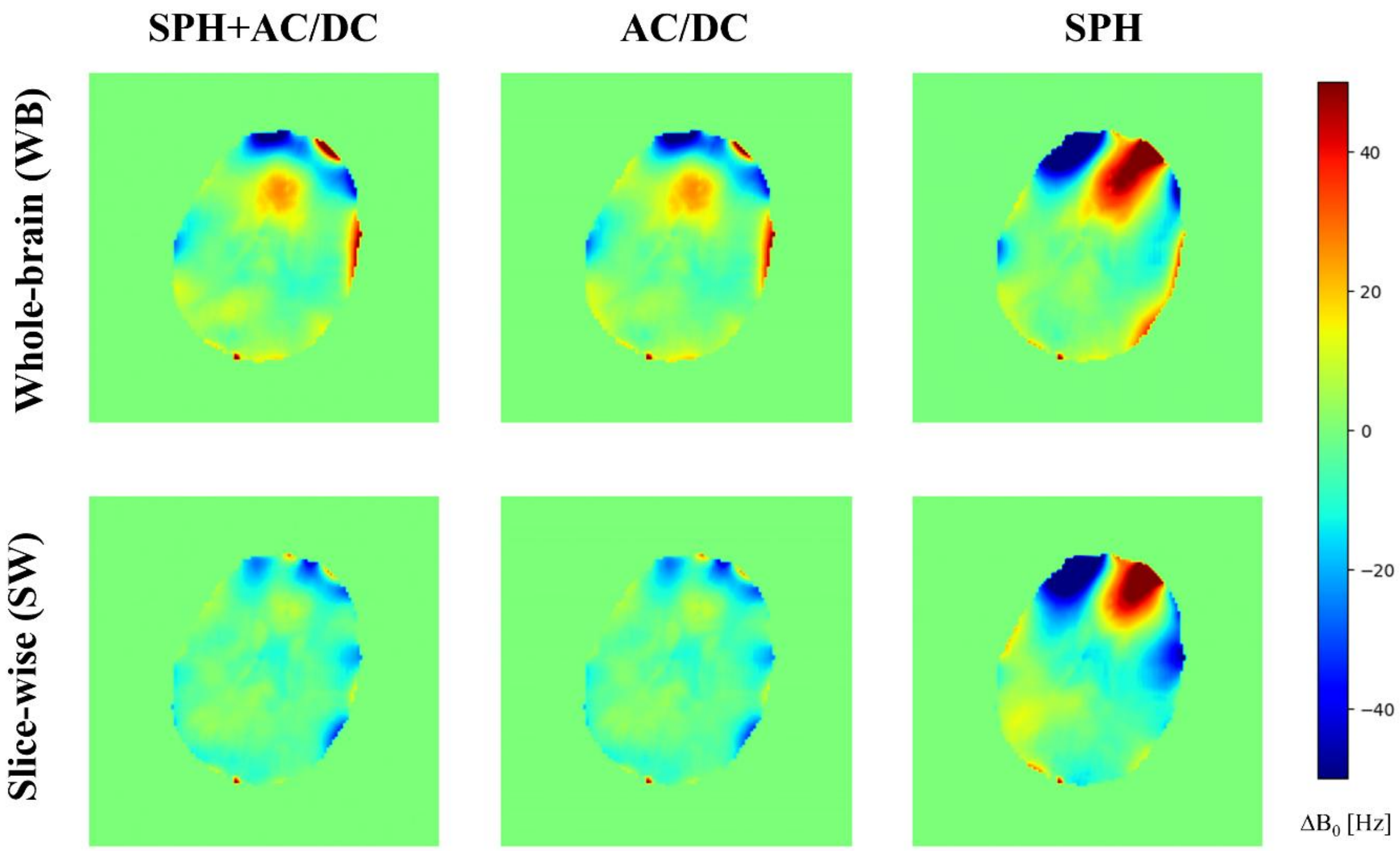

Figure S3. Representative B_0 field maps after RT-DL shimming in whole-brain (WB, top) and slice-wise (SW, bottom) modes for combined SPH+AC/DC, AC/DC, and SPH (left to right). AC/DC-based approaches reduce inhomogeneities compared to SPH, with minimal differences between AC/DC and combined SPH+AC/DC.

A quantitative comparison of the B_0 field maps after real-time shimming is shown in Figure S4. In WB shimming mode, both configurations involving AC/DC coils yielded lower standard deviation of the B_0 field than SPH alone. The median standard deviation was 23.31 Hz for combined SPH+AC/DC, 23.91 Hz for AC/DC alone, and 30.32 Hz for SPH, with both AC/DC-containing approaches showing significant improvements over SPH ($p < 0.001$).

In the SW mode, the benefits of AC/DC-based shimming were even more pronounced. Median standard deviations were 10.81 Hz for combined SPH+AC/DC, 11.94 Hz for AC/DC alone, and 18.85 Hz for SPH alone, again with significant differences between both AC/DC-containing approaches and SPH ($p < 0.001$). No significant difference was observed between combined SPH+AC/DC and AC/DC alone in neither WB or SW mode.

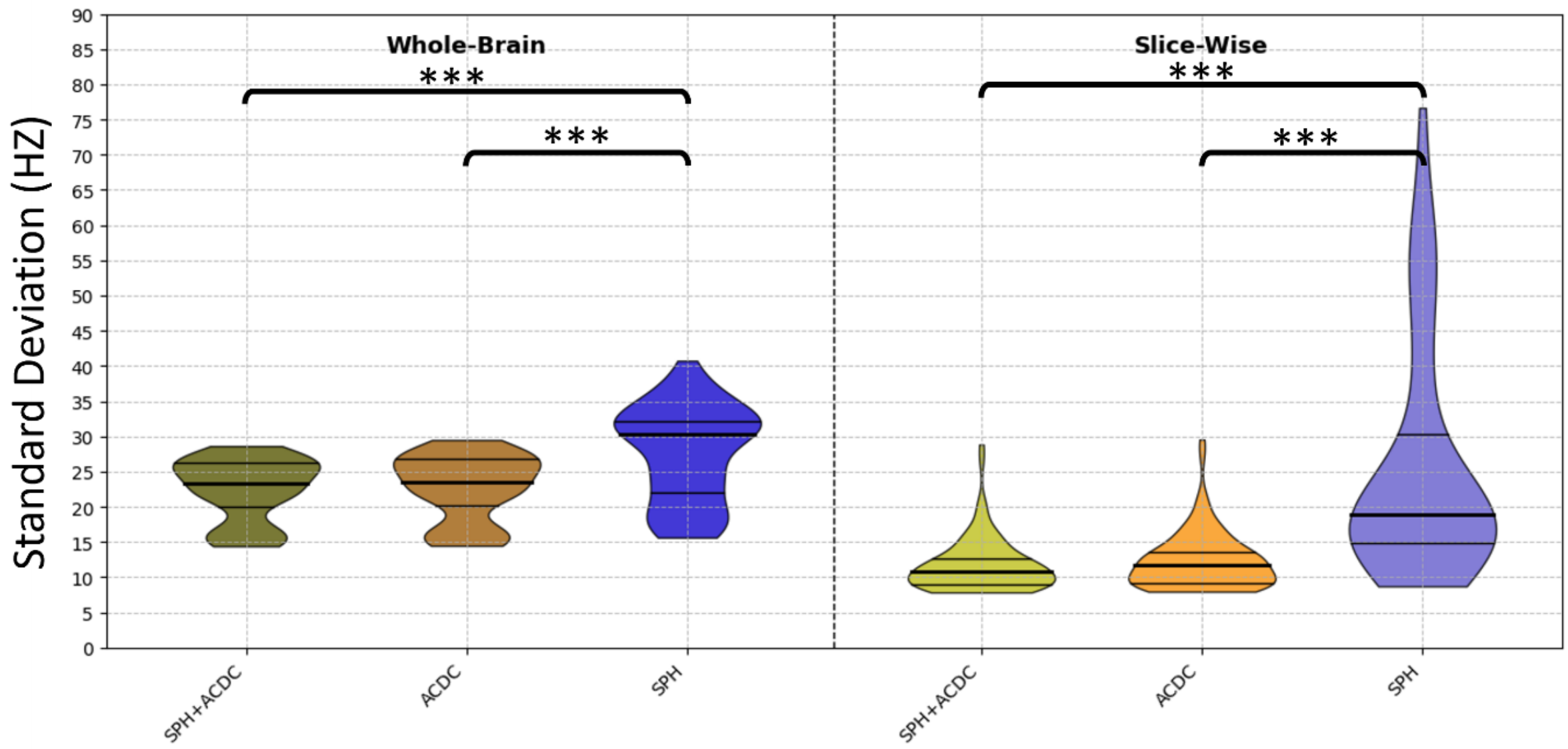

Figure S4. Distribution of the standard deviation of the B_0 field after deep-learning-based real-time shimming for different shim configurations (SPH, AC/DC, and combined SPH+AC/DC) in whole-brain (WB) and slice-wise (SW) modes. Violin plots illustrate the distribution across all 92 test positions, with median and interquartile ranges indicated. Both AC/DC and combined SPH+AC/DC configurations show significantly lower B_0 variability compared to SPH alone (***, $p < 0.001$). No significant difference is observed between AC/DC and combined SPH+AC/DC. The results demonstrate improved field homogeneity when incorporating AC/DC coils, particularly in the SW setting.

Overall, these results indicate that dynamic adjustment of AC/DC coils is the main contributor to the improvement in B_0 homogeneity, while the additional adjustment of SPH terms does not provide a measurable benefit beyond AC/DC-based shimming in this setting. This highlights that, although higher-order shimming can in principle improve field homogeneity, motion-induced B_0 variations require rapid updates, for which AC/DC coils provide a practical and efficient solution for real-time shimming.
